# Minimal Kinetic Model of Direct Air Capture of CO_2_ by Supported Amine Sorbents in Dry and Humid Conditions

**DOI:** 10.1021/acs.iecr.3c04535

**Published:** 2024-03-19

**Authors:** Hongjun Liu, Hongfei Lin, Sheng Dai, De-en Jiang

**Affiliations:** † Department of Chemical and Biomolecular Engineering, Vanderbilt University, Nashville, Tennessee 37235, United States; ‡ The Gene and Linda Voiland School of Chemical Engineering and Bioengineering, Washington State University, Pullman, Washington 99164, United States; ∥ Chemical Sciences Division, Oak Ridge National Laboratory, Oak Ridge, Tennessee 37831, United States; ⊥ Department of Chemistry, University of Tennessee, Knoxville, Tennessee 37996, United States

## Abstract

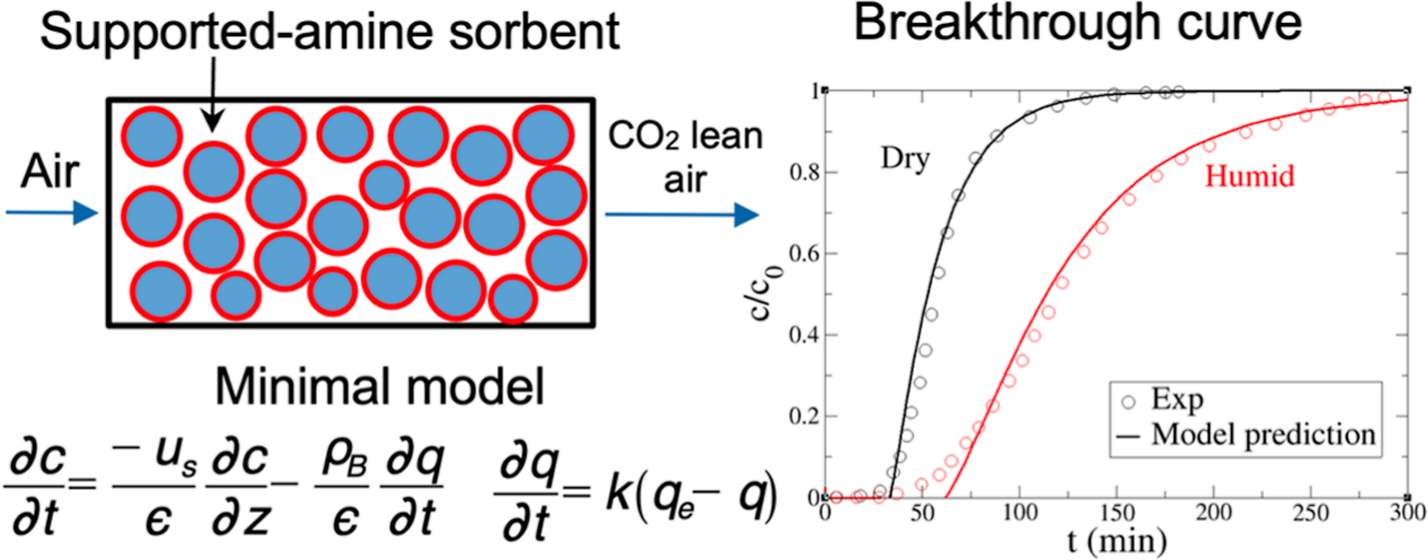

Dilute concentration (∼400 ppm) and humidity are two important factors in the direct air capture (DAC) of CO_2_ by supported sorbents. In this work, a minimal DAC CO_2_ adsorption-kinetics model was formulated for supported amine sorbents under dry and humid conditions. Our model fits well with a recent DAC experiment with supported amine sorbent in both dry and humid conditions. Temperature and flow rate effects on breakthrough curves were quantitatively captured, and increasing temperature led to faster CO_2_ adsorption kinetics. Moisture was shown to broaden the breakthrough curve with slower CO_2_ adsorption kinetics but significantly improve the uptake capacity. The present minimal model provides a versatile platform for kinetic modeling of the DAC of CO_2_ on supported amine and other chemisorption systems.

## 1 Introduction

Direct air capture (DAC) of CO_2_ is an important solution to control CO_2_ concentration in the atmosphere in order to mitigate climate change due to the increasing CO_2_ level.^1−3^ The DAC process usually involves the use of liquid or solid sorbents to selectively capture CO_2_ from the air via chemisorption, followed by sorbent regeneration via a temperate/pressure swing to release concentrated CO_2_ for storage or utilization. Supported amine sorbents have attracted significant attention for DAC, owing to their high adsorption capacity, excellent selectivity, beneficial coadsorption with moisture, and relatively mild energy requirements for sorbent regeneration.^4−10^ Physically impregnated or chemically grafted amine groups onto porous supports such as mesoporous silica, alumina, carbon, and metal–organic frameworks have been reported.^11−14^ The capture capacity is dictated by the reaction stoichiometry of amine groups with CO_2_ in dry or humid conditions, the temperature, and the loading of amines in the porous supports.^9,15−18^

The dilute concentration of CO_2_ in ambient air at about 400 ppm is one major challenge for DAC, requiring a strong thermodynamic driving force for its sorption. Most of the experimental and modeling work on amines focused on much higher CO_2_ partial pressures.^6,19−22^ Enhanced CO_2_ adsorption in the presence of H_2_O has been widely observed in amine adsorbents.^2,23−25^ Moreover, other chemisorption systems using superbase-derived ionic liquids have been developed.^26,27^ Although researchers have turned their attention to DAC conditions,^8,28,29^ how sorbent properties and H_2_O affect CO_2_ adsorption at DAC conditions is not well understood from a modeling perspective.^30−32^ On the experimental side, Sayari,^33^ Jones,^1^ and many others have extensively examined amine-functionalized mesoporous silica for DAC. The effect of water on CO_2_ adsorption has been recently reviewed by Sayari and co-workers,^10^ while Jones and co-workers have investigated many different types of porous supports loaded with poly(ethylenimine) (PEI).^34,35^ Molecular simulations at both the molecular mechanical and quantum mechanical levels have been employed to understand the DAC of CO_2_ and its interaction and reaction with amines as well.^36−39^

A few recent kinetic modeling efforts have examined carbon capture under the DAC conditions. Stampi-Bombelli et al. designed and analyzed temperature-vacuum swing adsorption cycles for DAC.^31^ Elfving and Sainio developed a detailed and new kinetic approach to model CO_2_ adsorption from humid air in amine-functionalized resins based on their experimental measurements.^32^ Young et al. proposed two different models to consistently describe H_2_O and CO_2_ coadsorption and to investigate their effects on DAC with amine-functionalized polymer-bead sorbents; they further performed process optimization for the DAC cycles.^40^ Overall, these very recent models are geared toward DAC process optimization, but for many researchers who are discovering and testing new porous sorbents at the benchtop scale, a simpler kinetic model that can fit the measured breakthrough curves well at various flow, temperature, and humid conditions would be more desirable to quickly evaluate the key thermodynamic and kinetic factors.

In this work, we develop a simple kinetic model of the DAC of CO_2_ by supported amine adsorbents to examine the effects of relevant parameters on breakthrough curves in dry and humid conditions in a packed bed and to help researchers quickly identify important thermodynamic and kinetic factors that can be used for further improvements of the DAC performances of amine-functionalized sorbents and other chemisorption systems. Below, we first describe our modeling approach and considerations and then validate our minimal model by comparing it to the literature. Next, we performed a parametric study to explore the effect of different parameters on breakthroughs in dry and humid conditions. Finally, we model a recent experimental DAC work to provide physical insights into the adsorption kinetics.

## 2 Methods

In the DAC process, a trace level of CO_2_ is adsorbed from a nonadsorbing carrier so that a single component model applies under dry conditions and a binary model under humid conditions. The mass transfer between gas and solid phases includes three resistances (external film, macropore, and micropore) and can be depicted by a linear driving force (LDF) model, where a lumped uptake rate constant is used to take various resistances into account.^41^ The LDF model is conceptually simple, computationally efficient, and therefore widely used in the literature.^21,42^ Momentum balance in the mobile gas phase includes viscous and kinetic terms; in a lab scale setup, constant gas velocity and negligible pressure drop can be assumed due to the short column length and low flow rate. Energy balance consists of three heat transfer equations for the gas phase, solid phase, and column wall. Local thermal equilibrium is assumed between gas and solid phases and also between the wall and ambient environment so that only one energy balance is needed for the whole packed bed system.

The mass balance equation in the gas phase is given as follows
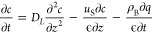
1where the three terms on the right-hand side are dispersion, convection, and adsorption on supported adsorbents, respectively; *c* is the gas concentration (mol/m^3^), *D*_L_ the axial dispersion coefficient (m^2^/s), *u*_s_ the superficial velocity (m/s), ρ_B_ the adsorbent bulk density (kg/m^3^), ϵ the bed void fraction (dimensionless), and *q* the adsorbate concentration (mol/kg). The LDF model (eq 2) was used to describe adsorption kinetics
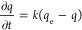
2where *k* (s^–1^) is the uptake rate constant (determined by optimizing the solution to the LDF model against the experimental breakthrough) and *q*_e_ (mol/kg) is the equilibrium adsorption concentration. *q*_e_ was determined by fitting the equilibrium experimental isotherms to the Toth adsorption model (eq 3) with temperature-dependent parameters *b* (atm^–1^), *n*_s_ (mol/kg), and *t*_T_ (dimensionless) expressed in eqs 4–6, respectively, where *n*_s0_ (saturation adsorption), *b*_0_ (adsorption affinity), Δ*H*_0_ (J/mol, heat of adsorption), and *t*_T0_ (Toth exponent) were optimized through nonlinear fitting to the experimental isotherm data. The amine-loading in the sorbent was not explicitly described in our model; instead, it was captured indirectly by the Toth parameters.
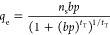
3
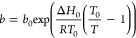
4
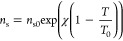
5
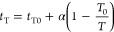
6

Similarly, the heat balance equation (eq 7) consists of dispersion, convection, adsorption, and wall heat transfer terms
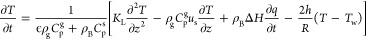
7where *C*_p_ is the specific heat capacity (J kg^–1^ K^–1^), *K*_L_ axial thermal conductivity (W m^–1^ K^–1^), *h* wall heat transfer coefficient (W m^–2^ K^–1^), and *R* column radius (m). Initial conditions used were *c* = 0, *q* = 0, and *T* = *T*_0_ at *t* = 0, and boundary conditions were *c* = *c*_0_ and *T* = *T*_0_ at *z* = 0, and ∂*c*/∂*z* = 0 and ∂*T*/∂*z* = 0 at *z* = *L*.

The Guggenheim-Anderson-de Boer (GAB) model was used to describe H_2_O uptake (*q*_2_) for both single component and binary systems as a function of relative humidity (ϕ, dimensionless)
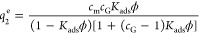
8where *c*_m_ (mol/kg), *c*_G_ (dimensionless), and *K*_ads_ (dimensionless) are model parameters. The saturated vapor pressure can be calculated by the empirical Buck equation
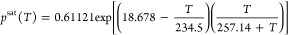
9where *p*^sat^ is in the unit of kPa and *T* of °C.

Method of lines was applied to solve PDEs numerically. *N* grid points were discretized on a length scale. The second-order centered difference approximation formula was used to estimate the first and second derivatives. Each PDE was subsequently converted to *N* ODEs. The resulting ODEs were simultaneously solved by the ode15s solver in MATLAB to obtain the profiles of the gas concentration *c*(r,t), adsorbed gas concentration in adsorbents *q*(r,t), and gas temperature *T*(r,t). The ode15s solver uses the variable order method to solve ODEs, which dynamically chooses discretization methods of different orders and sets the time grid automatically; it also uses the variable step-size method.

## 3 Results and Discussion

### 3.1 Model Benchmark at the Flue-Gas Conditions

**Figure 1:**
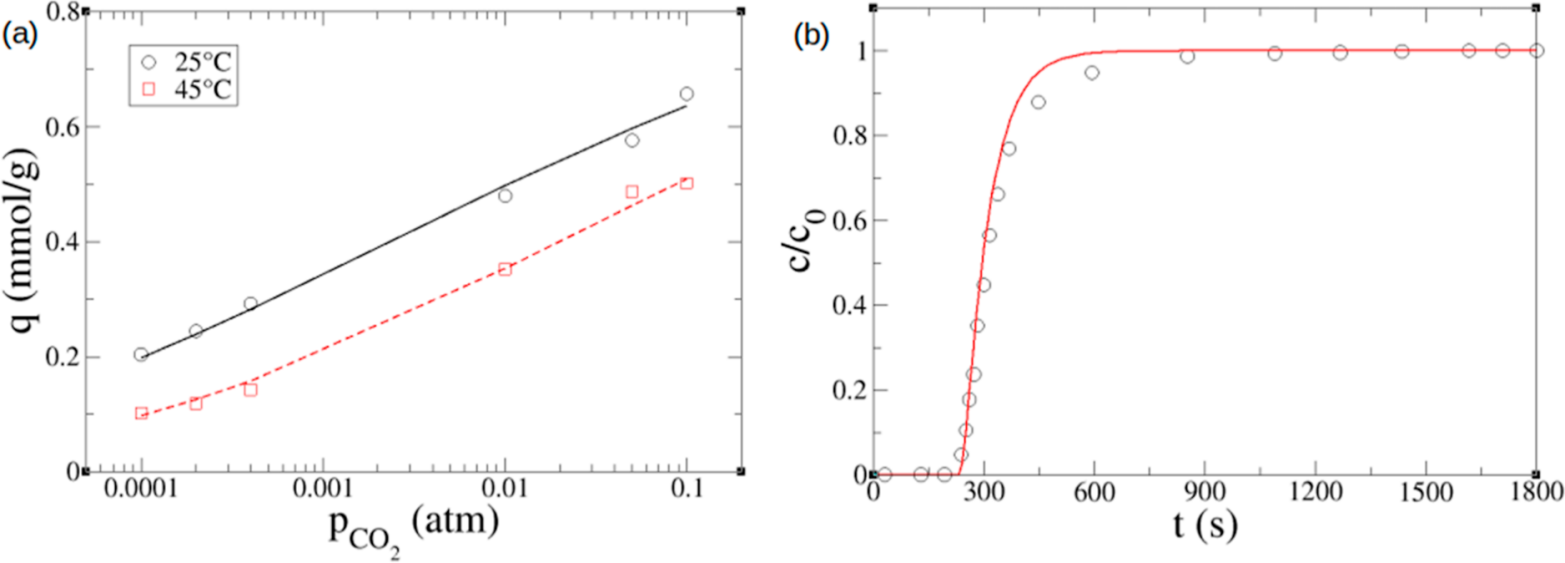
(a) Toth model fit (line) using eq 3 to the experimental isotherm data (symbol) in amine-functionalized SBA-15 adsorbent (via 3-aminopropylsilyl groups) at two different temperatures (experimental isotherms were measured with a thermogravimetric analyzer for sorbent pellets of 150–550 μm in size).^21^ (b) Comparison between model prediction (line) and experiment (symbol) of breakthrough profiles of the amine-functionalized SBA-15 sorbent (0.1 atm CO_2_, 25 °C, dry).^21^

To validate our implementations, we chose to reproduce the breakthrough curves reported in the amine-functionalized SBA-15 silica adsorbent for the flue-gas conditions (0.1 atm CO_2_).^21^ The experimental equilibrium adsorption data were fitted to a Toth isotherm, and the quality of the fitting is very good (Figure 1a). Fitting parameters (*n*_s_ = 0.97 mmol/g, *b*_0_ = 8.3 × 10^5^ atm^–1^, Δ*H*_0_ = 84 kJ/mol, and *t*_T_ = 0.21) are comparable to the previously reported values (*n*_s_ = 1 mmol/g, *b*_0_ = 3 × 10^5^ atm^–1^, Δ*H*_0_ = 75 kJ/mol, and *t*_T_ = 0.22).^21^ More importantly, excellent agreement was achieved between our model prediction and the experimental breakthrough profile under the flue gas conditions (Figure 1b). In addition, it has been shown that simple isothermal models provide an accurate description of the mass transport behavior in the adsorption column at the benchtop scale,^21^ so here, we also adopt an isothermal model to study the breakthrough curves at even lower CO_2_ concentrations such as DAC conditions (400 ppm of CO_2_). The mass balance equation was further simplified by dropping out the second derivative dispersion term, since the convection term is dominant. The LDF kinetic model and Toth isotherm were used to complement the following PDE for the numerical solution. This simplified version (eq 10) is the minimal model for DAC CO_2_ adsorption.
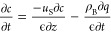
10

### 3.2 Parametric Study at DAC Conditions

**Figure 2:**
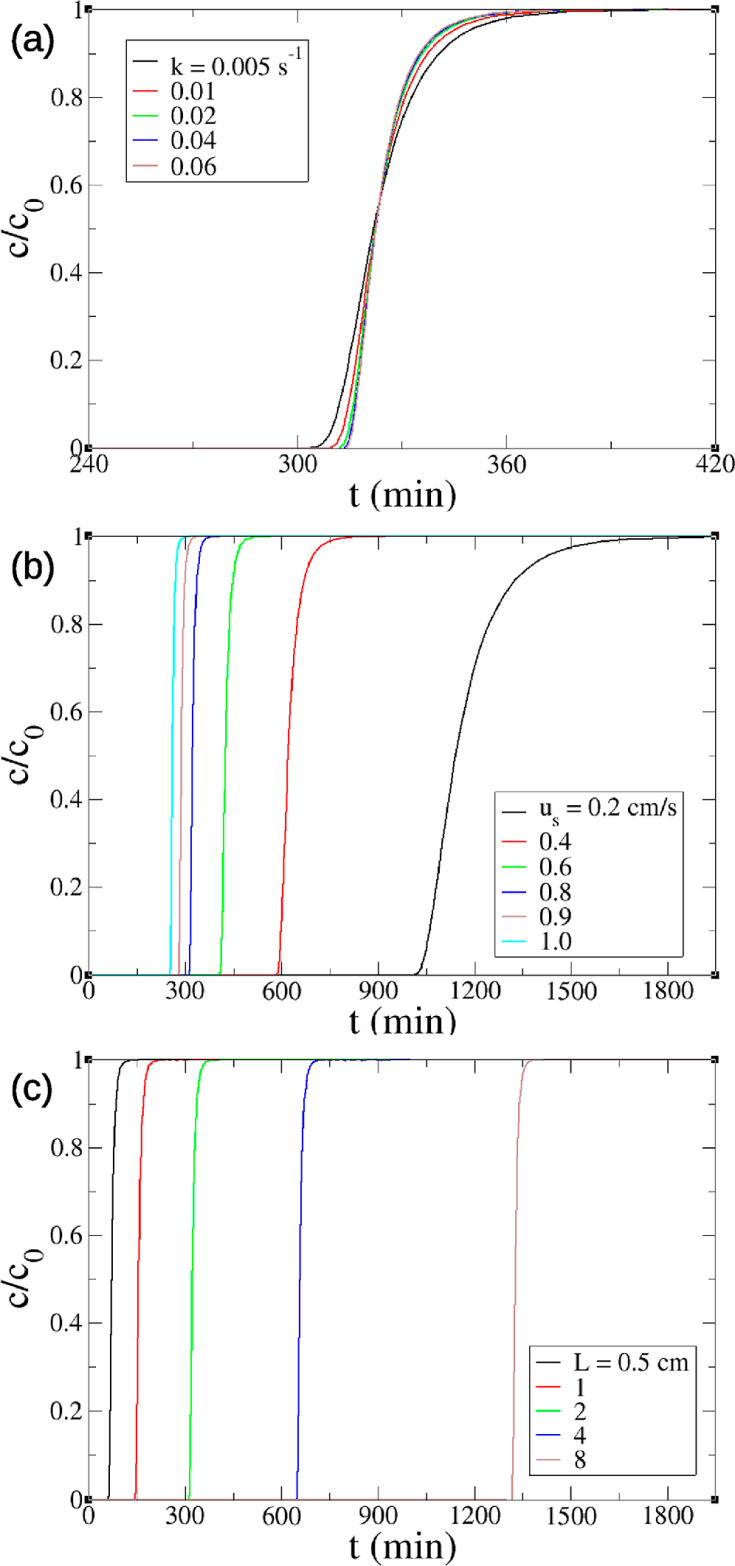
Breakthrough curves of the minimal model at DAC conditions (400 ppm of CO_2_, 25 °C, dry) with varying parameters: (a) uptake rate constant k; (b) superficial velocity *u*_s_; and (c) column length *L*.

**Figure 3:**
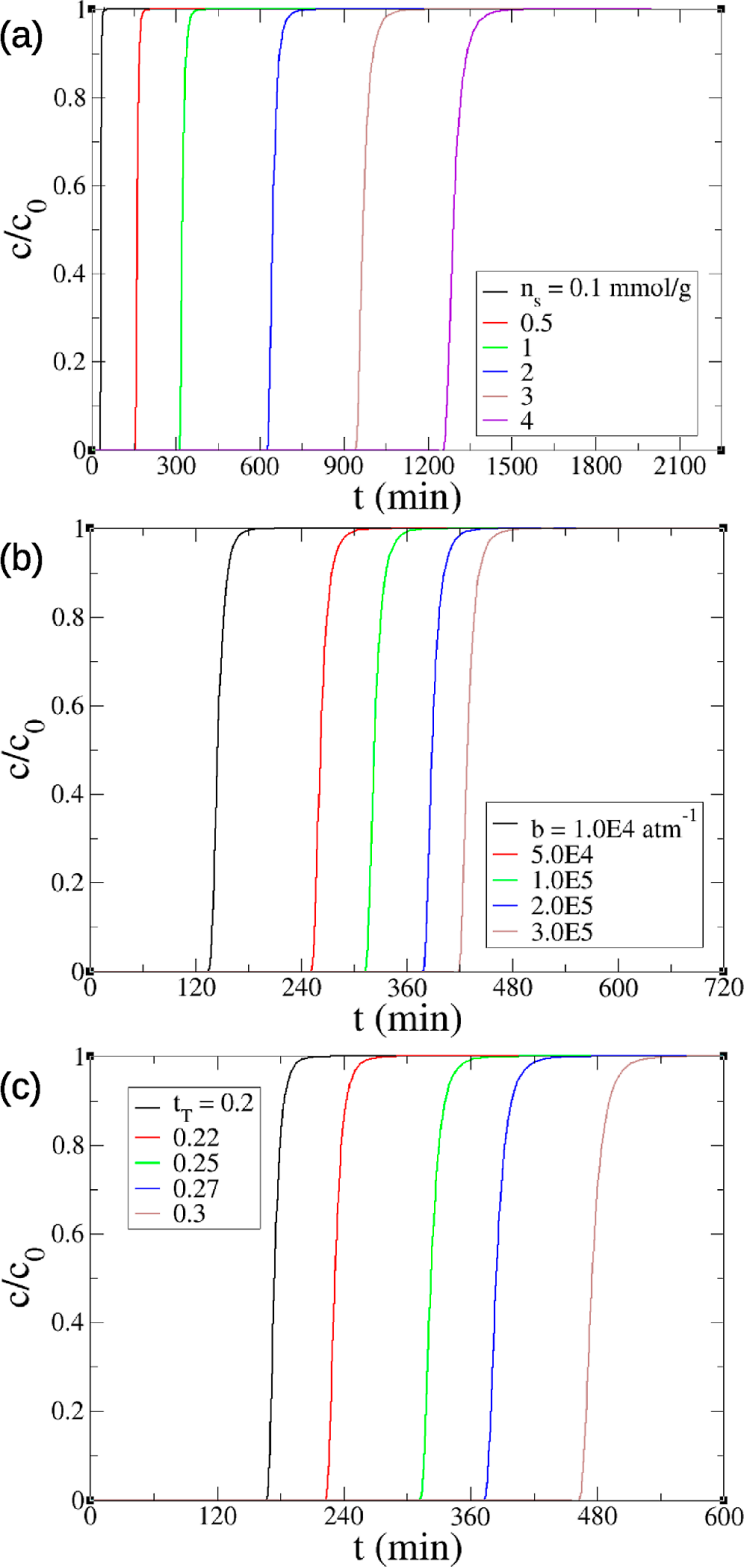
Effects of Toth model parameters on the breakthrough curves of the minimal model at DAC conditions (400 ppm of CO_2_, 25 °C, dry): (a) saturation adsorption *n*_s_; (b) adsorption affinity *b*; and (c) Toth exponent *t*_T_.

Using our minimal kinetic model, we examined the effects of *k*, *u*_s_, and *L* on the breakthrough curves at DAC conditions. Uptake rate constant *k*, a lumped term combining mass diffusion rate and CO_2_ adsorption rate, dictates the breakthrough curve shape.^43^ Superficial velocity *u*_s_ and column length *L* are important controlling parameters for unit operation of the packed adsorption bed. The Toth isotherm and modeling parameters used in the simulations are listed in Tables 1 and 2, respectively. Several qualitative trends can be clearly identified. The larger uptake rate constant *k* leads to a steeper breakthrough (Figure 2a) due to the faster adsorption kinetics. Larger gas velocity *u*_s_ leads to faster and steeper breakthroughs (Figure 2b) because a larger gas velocity or flow rate gives rise to a stronger convection contribution, causing a larger concentration difference and, hence, greater driving force. Figure 2c shows that the longer the packed column, the slower the breakthrough because the adsorbent bed has a larger adsorption capacity. The effects of Toth model parameters on the breakthrough curves are shown in Figure 3. The larger *n*_s_, *b*, and *t*_T_ lead to a slower breakthrough due to the larger equilibrium adsorption *q*_e_, since it takes more time for the packed column to get saturated as the equilibrium adsorption capacity increases.

**Table 1:** Toth and Modified Toth Isotherm Parameters for CO_2_ and GAB Isotherm Parameters for H_2_O Used in the Parametric Study at DAC Conditions

	parameters	values
Toth for CO_2_	*b*	1.0 × 10^5^ atm^–1^
	*n*_s_	1.0 mmol/g
	*t*_T_	0.25
modified Toth for CO_2_ with H_2_O	γ	6.0 × 10^–3^ g/mmol
	β	25 g/mmol
GAB for H_2_O	*c*_m_	35 mmol/g
	*c*_G_	0.15
	*K*_ads_	0.51

**Table 2:** Preset Input Conditions Used in Parametric Study of Kinetic Modeling at DAC Conditions

parameters	values
bed void fraction ϵ	0.5
adsorbent bulk density ρ_B_	0.5 g/cm^3^
bed length *L*	2 cm
superficial velocity *u*_S_	0.8 cm/s
CO_2_ uptake rate constant *k*_1_	0.02 s^–1^
H_2_O uptake rate constant *k*_2_	0.03 s^–1^
CO_2_ feed concentration *c*_10_ of 400 ppm	1.6 × 10^–5^ mmol/cm^3^
relative humidity ϕ	20–50%
H_2_O feed concentration *c*_20_ at ϕ = 40%	2.6 × 10^–4^ mmol/cm^3^

### 3.3 Humidity Effect

Water vapor is ubiquitous in ambient air, and the water concentration is significantly higher than the CO_2_ concentration; therefore, including the H_2_O component in the model mixture to properly consider cooperative CO_2_ coadsorption with moisture is essential. The air was modeled as a ternary mixture of CO_2_, H_2_O, and N_2_ at a constant composition of 400 ppm of CO_2_, with a 40% relative humidity of H_2_O and the remainder of N_2_. Note that N_2_ was used to model all inert carrier components in air, which have low or negligible affinity to amine adsorbents. So, the LDF (eq 2) and minimum model (eq 10) were applied to both CO_2_ (*c*_1_) and H_2_O (*c*_2_). To consider CO_2_ coadsorption with moisture, a modified Toth model with enhanced adsorption affinity and saturated adsorption capacity was applied.^31^ When H_2_O is present in the amine adsorbents, *n*_s_ and *b* in the Toth isotherm are updated accordingly as a function of temperature, CO_2_ partial pressure, and adsorbed H_2_O quantity: *n*_s_ (*q*_2_) = *n*_s_ (1/(1 – γ*q*_2_)) and *b*(*q*_2_) = *b*(1 + β*q*_2_), where γ and β are positive parameters (Table 1). H_2_O isotherm was described by the GAB model,^44^ whose parameters are also shown in Table 1.

**Figure 4:**
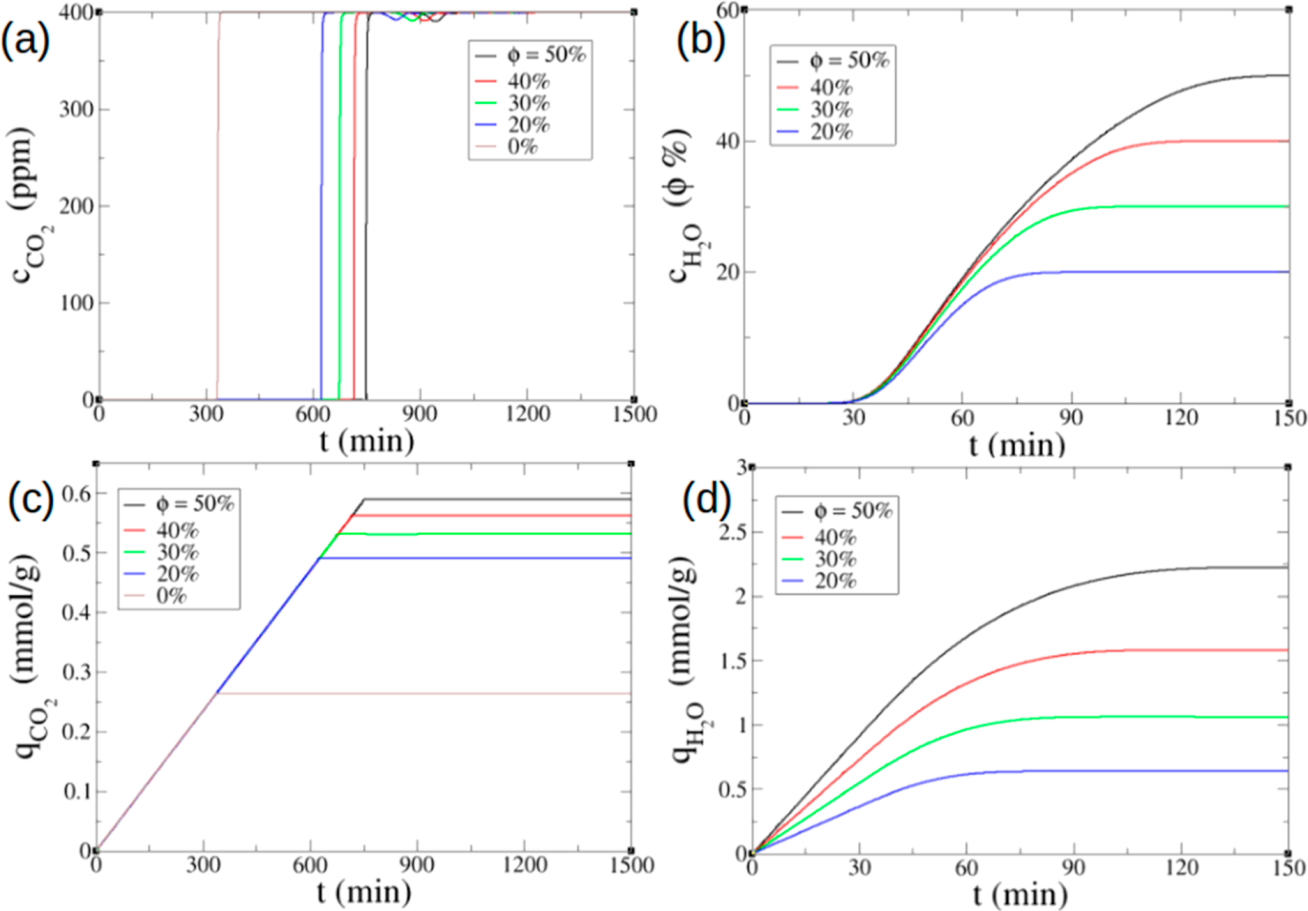
Effect of relative humidity (ϕ) on DAC (400 ppm of CO_2_, 25 °C) simulated with the minimal model for the packed bed: (a) CO_2_ breakthrough curves; (b) H_2_O breakthrough curves; (c) CO_2_ dynamic adsorption capacity; and (d) H_2_O dynamic adsorption capacity.

Figure 4 shows the effect of relative humidity (ϕ) on DAC. One can see that the CO_2_ breakthrough curves shift to longer times when the humidity level increases (Figure 4a) due to the enhanced CO_2_ adsorption capacity with moisture. The improved capacity in humid conditions, captured by the modified Toth isotherm with an empirical enhancement factor, is due to the opening of an additional reaction pathway which allows bicarbonate formation from water, amine group, and CO_2_.^45^ In dry conditions, CO_2_ breaks through the adsorption column around 330 min, while 630 min at ϕ = 20% and 750 min at ϕ = 50% (Figure 4a). In comparison, H_2_O concentration at the outlet reaches the feed saturated level much faster (Figure 4b): it hardly changes after 80 min at ϕ = 20% and 140 min at ϕ = 50%. The dynamic adsorption capacity of the bed as a function of time can be used to estimate the breakthrough uptake given the breakthrough time, which was defined as the time corresponding to an arbitrary ratio, e.g., *c*/*c*_0_ = 0.1, at the breakthrough curve. For instance, the breakthrough CO_2_ uptake at ϕ = 20% is about 0.49 mmol/g (Figure 4c) and H_2_O uptake is around 0.62 mmol/g (Figure 4d).

### 3.4 Application of Our Minimal Model to a Recent DAC Experiment

**Figure 5:**
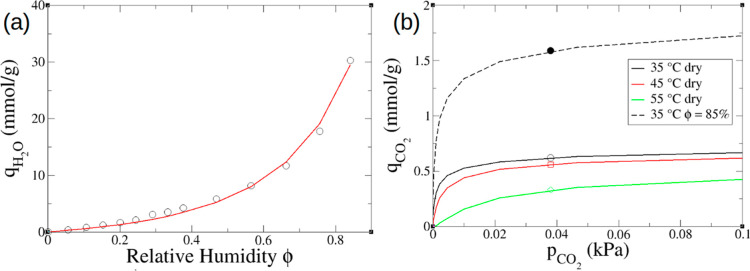
Application of our minimal model to the experimental data of DAC by PEI-impregnated silica fiber sorbents in humid conditions (380 ppm of CO_2_): (a) fitted H_2_O equilibrium uptake against relative humidity (line) in comparison with the experiment (symbol);^46^ (b) fitted CO_2_ isotherms (line) at different temperatures in comparison with the experiment (symbols).^46^

We applied the minimal model to simulate a recent experiment where the authors studied temperature, flow rate, and humidity effects on breakthrough curves of PEI-impregnated silica fiber sorbents with a 380 ppm of CO_2_ feed.^46^ To begin with, the water isotherm data was fitted to the GAB model with *c*_m_ = 26 mmol/g, *c*_G_ = 0.22, and *K*_ads_ = 0.84 (Figure 5a). The pseudoequilibrium CO_2_ capacities obtained from the breakthrough experiment were used to approximate the equilibrium data. The Toth model with the following parameters (*T*_0_ = 308 K, *n*_s0_ = 0.81 mmol/g, *b*_0_ = 6.2 × 10^3^ kPa^–1^, *t*_T0_ = 0.40, Δ*H*_0_ = 210 kJ/mol, and χ = 6.6, α = 10.8) can well capture temperature dependence of the CO_2_ isotherm. An empirical enhancement factor of *e*^1.11ϕ^ was found to describe improved CO_2_ coadsorption with moisture; the resulting single (dry) and binary (humid) CO_2_ isotherms present a realistic representation of experimental data (Figure 5b).

**Figure 6:**
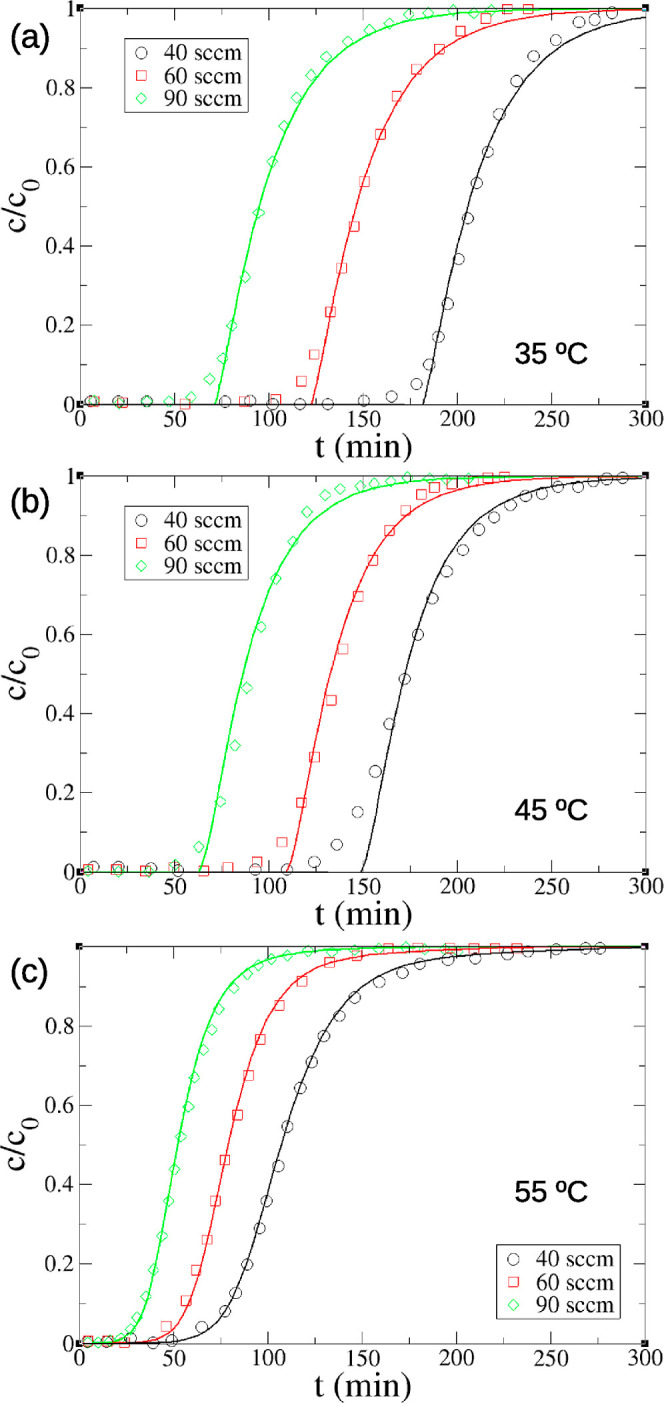
Comparison of dry DAC breakthrough curves (380 ppm of CO_2_) between modeling prediction (line) and experiment^46^ (symbol) at different feed flow rates and temperatures: (a) 35; (b) 45; and (c) 55 °C. The optimized uptake rate constants are 0.04 min^–1^ at 35 °C, 0.048 min^–1^ at 45 °C, and 0.1 min^–1^ at 55 °C (1 sccm = 1 cm^3^ STP/min; STP is the standard condition of 0 °C and 1 bar).

We further simulated the effects of temperatures and flow rates on the breakthrough curves and found excellent agreement between the modeling prediction and experiment (Figure 6). As temperature increases, the difference in breakthrough diminishes as a function of flow rate. Fitting the optimized uptake rate constants (0.04 min^–1^ at 35 °C, 0.048 min^–1^ at 45 °C, and 0.1 min^–1^ at 55 °C) at the dry DAC conditions to the Arrhenius equation leads to an adsorption activation energy of *E*_a_ = 38 ± 14 kJ/mol, which is in reasonable agreement with the measured activation energy of DAC by PEI-functionalized silica sorbent in the similar conditions (28.6 kJ/mol).^22^ It is generally believed that increasing the temperature improves CO_2_ diffusion through the aggregated amine phase inside the pores of solid support, thereby facilitating CO_2_ adsorption.^20^ Yet, the favorable kinetics with increasing temperature is not significant enough to overcome the dominant thermodynamic trend; that is, increasing temperature lowers CO_2_ uptake capacity. The overall effect explains the general trend of breakthrough and pseudoequilibrium uptakes observed in the breakthrough experiment. One can see from Table 3 that both the experimental breakthrough (*q*_b_) and pseudoequilibrium (*q*_e_) capacities decrease with temperature, while the breakthrough capacity (*q*_b_) also decreases with the flow rate.^46^ In higher flow rates, CO_2_ molecules have less residence time in the packed bed to reach adsorption equilibrium, leading to sharper breakthrough curves and lower breakthrough capacities. Table 3 shows that the predicted breakthrough and pseudoequilibrium capacities agree well with the experimental values.

**Table 3:** Comparison of Prediction and Experiment^46^ of Breakthrough (*q*_b_) and Pseudoequilibrium (*q*_e_) Capacities at 5% *c*/*c*_0_ and 95% *c*/*c*_0_, Respectively, at DAC Conditions (380 ppm of CO_2_, Dry)

	*q*_b_ (mmol/g): prediction/experiment			*q*_e_ (mmol/g): prediction/experiment		
	40sccm	60sccm	90sccm	40sccm	60sccm	90sccm
35 °C	0.53/0.51	0.50/0.48	0.44/0.44	0.62/0.61	0.62/0.61	0.62/0.62
45 °C	0.47/0.41	0.43/0.37	0.39/0.36	0.56/0.55	0.56/0.56	0.56/0.56
55 °C	0.21/0.19	0.19/0.13	0.16/0.08	0.33/0.32	0.33/0.33	0.33/0.33

**Figure 7:**
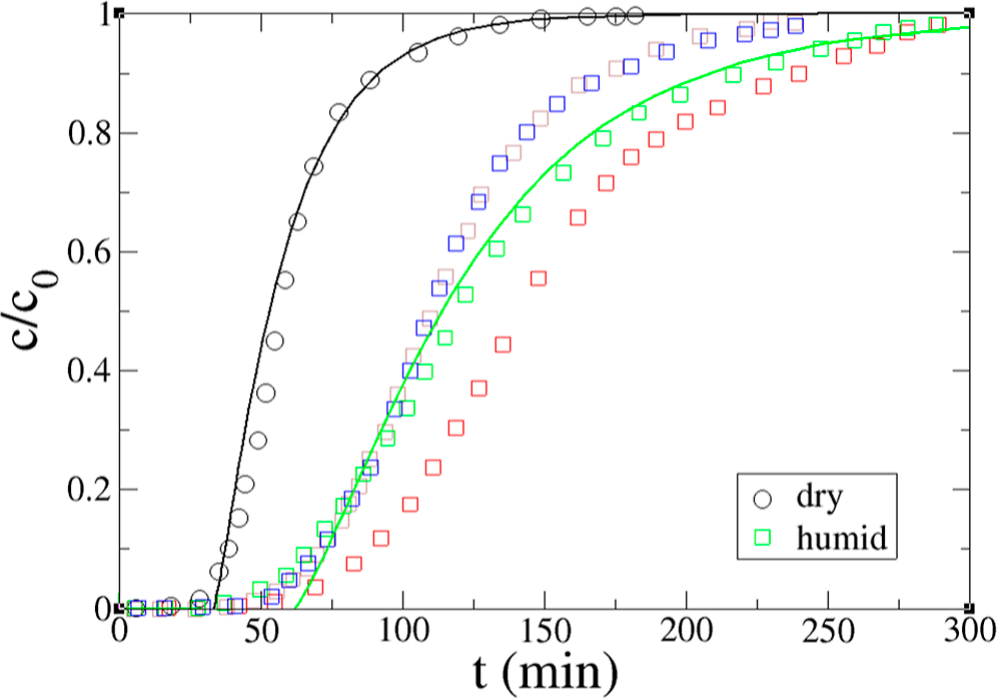
Comparison of modeling prediction (line) and experiment (symbol) of dry and humid 380 ppm of CO_2_ breakthrough curves at 35 °C and 200 sccm. In the experiment,^46^ multiple independent runs (denoted by open squares of different colors) of humid CO_2_ were performed.

We further investigated the effect of humidity on the CO_2_ breakthrough at 85% relative humidity. The predicted breakthrough curves are in good agreement with those from the experiment (Figure 7). A significant slowdown of the CO_2_ adsorption kinetics was found. The CO_2_ uptake rate constant decreases from 0.04 min^–1^ in dry conditions to 0.02 min^–1^ in humid conditions, while the H_2_O uptake rate constant is much faster at 0.36 min^–1^. Improved CO_2_ adsorption upon the addition of moisture is generally explained by the formation of additional carbonate/bicarbonate species and enhanced amine accessibility due to facile CO_2_ diffusion.^10^ Our modeling result suggests that such improvements do not necessarily lead to faster CO_2_ adsorption kinetics. The slower kinetics of CO_2_ adsorption under humid conditions could be due to slower gas diffusion in the sorbent or a slower reaction with the amines in the presence of water. Our current model lumped those factors into one kinetic parameter, the CO_2_ uptake rate constant (*k*), and could not differentiate their contributions. In terms of capacity, the predicted breakthrough and pseudoequilibrium capacities are in good agreement with the experiment in both dry and humid conditions (Table 4). The overall effects of various conditions are summarized in Table 5.

**Table 4:** Comparison of Prediction and Experiment^46^ of Breakthrough (*q*_b_) and Pseudoequilibrium (*q*_e_) Capacities at 5% *c*/*c*_0_ and 95% *c*/*c*_0_, Respectively, at Dry and Humid Conditions at 35 °C and 200 sccm (380 ppm of CO_2_)

	*q*_b_ (mmol/g): prediction/experiment	*q*_e_ (mmol/g): prediction/experiment
dry	0.37/0.36	0.62/0.62
humid	0.80/0.64	1.60/1.60

**Table 5:** Summary of the Effects of the Varying Conditions on the DAC Breakthrough Curves from Our Minimal Kinetic Model

conditions	effects
increasing uptake rate constant	steeper breakthrough
increasing flow rate	faster breakthrough, lower breakthrough capacity
increasing fixed bed length	slower breakthrough, higher breakthrough capacity
increasing equilibrium capacity	slower breakthrough, higher breakthrough capacity
increasing temperature	lower equilibrium capacity, faster adsorption kinetics, faster breakthrough, lower breakthrough capacity
humid CO_2_	larger equilibrium capacity, slower and broader breakthrough, higher breakthrough capacity

### 3.5 Limitations of Our Model

Despite its simplicity and utility, the minimal model that we have developed here has limitations due to the assumptions made. We summarize here the assumptions and limitations: (1) plug flow, constant gas superficial velocity, no axial dispersion; (2) isotherm model, in other words, heat transfer has an insignificant effect on the breakthrough curve in the benchtop scale (nonisotherm model with energy balance is necessary for DAC cycle process modeling and techno-economic analysis); (3) one-dimensional model, so gas concentration gradients only exist in the axial direction; (4) gas phase behaves as ideal gas; (5) negligible pressure drop due to the short column length and relatively low flow rate (pressure drop can be described by the Ergun equation if needed); (6) only CO_2_ and H_2_O adsorption are considered, and all others are treated as nonadsorbing components; and (7) LDF adsorption kinetics model (more complex mass transfer resistance model could be applied to describe different mass transfer resistances from the gas phase to the solid sorbent phase).

## 4 Conclusions

CO_2_ under dry and humid DAC conditions was kinetically simulated using a minimal mathematical model. Parameters related to process operation, isotherm, and adsorption kinetics were varied to study the general effect on breakthrough curves. The larger uptake rate leads to a steeper breakthrough. Either the longer column or the slower gas velocity gives rise to a slower breakthrough. The larger equilibrium adsorption arising from the larger saturated adsorption or adsorption affinity or Toth exponent also leads to a slower breakthrough. The humidity effect on breakthrough curves and bed adsorption capacity were studied with the help of a modified Toth isotherm to properly consider enhanced CO_2_ coadsorption with moisture. Increasing relative humidity shifts the breakthrough curve to a longer time with a higher adsorption capacity of the bed. The implemented methodology was successfully applied to model a recent DAC experiment of supported amine sorbent under both dry and humid conditions. Temperature and flow rate effects on breakthrough were quantitatively captured. The CO_2_ uptake rate constantly increases with temperature. Moisture broadens the breakthrough curve with a significantly improved uptake capacity but slower CO_2_ adsorption kinetics. The minimal model developed in this work will be useful in understanding the thermodynamics and kinetics of the DAC of CO_2_ on supported amine and other chemisorption systems.
